# Divergent roles of OmpA family proteins in physiology, stress tolerance, and virulence of *Elizabethkingia miricola*

**DOI:** 10.1080/21505594.2026.2645897

**Published:** 2026-04-11

**Authors:** Fangyuan Liu, Fafa He, Zemao Gu, Ruixue Hu

**Affiliations:** a College of Fisheries/Shuangshui Shuanglü Institute, Huazhong Agricultural University, Wuhan, China; b Hubei Engineering Technology Research Center for Aquatic Animal Diseases Control and Prevention, Wuhan, China; c National Aquatic Animal Diseases Para-Reference Laboratory (HZAU), Wuhan, China; d Hubei Hongshan Laboratory, Wuhan, China

**Keywords:** *Elizabethkingia miricola*, OmpA family proteins, virulence, molecular targets

## Abstract

*Elizabethkingia* species are emerging opportunistic pathogens responsible for life-threatening nosocomial infections. Their intrinsic multidrug resistance severely limits effective antibiotic treatment, necessitating urgent development of novel therapeutic strategies. Among potential targets, outer membrane protein A (OmpA) family proteins have drawn increasing interest. However, the functional roles of OmpA family proteins in *Elizabethkingia* remain entirely uncharacterized, leaving their potential as targeted therapeutics unresolved. In this study, we identified a group of putative OmpA family proteins in *Elizabethkingia miricola* strain FL160902 and systematically investigated the contributions of five OmpA members to bacterial physiology and virulence. Targeted gene deletions revealed marked functional divergence among these paralogs. Specifically, OmpA-1 and OmpA-3 were identified as master virulence determinants; their deletion resulted in impaired serum resistance, reduced cell adhesion capacity, and diminished *in vivo* lethality. Their deletion also induced extensive physiological dysregulation, including enhanced biofilm formation, increased surface hydrophobicity, and heightened susceptibility to oxidative stress. In contrast, deletions of *ompA*-2, *ompA*-4, and *ompA*-5 resulted only in partial attenuation of virulence traits and restricted changes to specific physiological phenotypes. Collectively, these findings establish a functional hierarchy within the OmpA proteins and position OmpA-1 and OmpA-3 as high-priority molecular targets for developing precision therapeutics against this life-threatening, multidrug-resistant pathogen.

## Introduction

*Elizabethkingia*, a genus of Gram-negative bacteria, has emerged as an opportunistic pathogen causing severe infections such as bacteremia, pneumonia, sepsis, and meningitis, particularly in immunocompromised individuals, neonates, and the elderly [1–3]. The genus currently comprises eight recognized species, with *Elizabethkingia meningoseptica*, *Elizabethkingia miricola*, and *Elizabethkingia anophelis* being of primary clinical relevance [4,5]. *E. miricola*, first isolated in 2003 from condensate within the Mir Space Station [6], has been increasingly implicated in life-threatening hospital-acquired infections [7–9]. Moreover, this species has been identified as the etiological agent of meningitis-like epidemics in frogs across both wild and captive populations, raising concerns about its potential as a zoonotic threat and broader public health risk [10–13]. *Elizabethkingia* species exhibit intrinsic resistance to a broad spectrum of clinically important antibiotics, severely limiting treatment options and contributing to high rates of therapeutic failure [14–16]. Consequently, there is an urgent need to develop novel drug targets and antibacterial strategies for the effective management of infections caused by these pathogens.

The identification of critical virulence determinants and the elucidation of their underlying molecular mechanisms are essential for discovering novel therapeutic targets. Several potential virulence factors have been identified in *Elizabethkingia* species, including capsular polysaccharide gene clusters, protein secretion systems, outer membrane proteins (OMPs), and iron/heme acquisition-related proteins [17,18]. Among these, the outer membrane protein A (OmpA) has garnered significant interest due to its well-established role in the virulence of various Gram-negative bacterial pathogens, making it a promising candidate for the development of new drugs and vaccines [19–21].

The OmpA family is a group of surface-exposed porin proteins abundantly expressed in the outer membranes of Gram-negative bacteria. Structurally, these proteins are defined by an N-terminal domain comprising an eight-stranded β-barrel embedded in the outer membrane, with four extracellular loops exposed to the external environment, and a C-terminal periplasmic domain that binds peptidoglycan to stabilize the cell envelope [22]. Functioning as nonselective transmembrane channels, OmpA proteins facilitate the passive diffusion of ions and small solutes across the outer membrane [23]. Beyond their fundamental role in maintaining bacterial homeostasis, OmpA family members serve as critical virulence factors, facilitating bacterial colonization and infection by mediating adhesion to and invasion of diverse host niches [24]. For instance, OmpA drives the adhesion to and invasion of central nervous system tissues in meningitic *Escherichia coli* [25,26], and facilitates mucosal colonization of intestinal, cervical, and respiratory epithelia in enteropathogenic *E. coli*, *Neisseria gonorrhoeae*, and *Haemophilus influenzae*, respectively [27–29]. Additionally, OmpA proteins enable bacteria to evade host defenses through multifaceted mechanisms. These include conferring serum resistance, as exemplified by the recruitment of factor H by *Acinetobacter baumannii* OmpA to inhibit complement activation [30]; enhancing tolerance to antimicrobial peptides in *Klebsiella pneumoniae* [31]; and protecting *Salmonella Typhimurium* against macrophage-derived nitrosative stress to ensure intracellular survival [32]. Given these pleiotropic roles in pathogenicity, OmpA has emerged as a compelling target for novel antimicrobial interventions. Emerging anti-virulence strategies, such as OmpA-specific peptide inhibitors that block host-cell adhesion and small-molecule agents that disrupt OmpA expression at transcriptional or post-transcriptional levels in *Acinetobacter baumannii*, have shown significant promise [33,34]. In contrast to these advances, the specific functional contributions of OmpA in *Elizabethkingia* remain poorly understood.

In this study, we identified and bioinformatically analyzed a group of putative OmpA proteins in *E. miricola* strain FL160902. To elucidate their functions, we generated isogenic single-gene knockout mutants and corresponding complementation strains for all *ompA* genes through homologous recombination. Subsequent phenotypic profiling revealed their roles in bacterial physiology, stress adaptation, and virulence. Our findings provide initial insights into the functional diversification of OmpA family proteins in *E. miricola*, advancing the understanding of OmpA-mediated pathogenicity and informing in the development of innovative therapeutic strategies against *Elizabethkingia* infections.

## Materials and methods

### Bacterial strains, plasmids, and culture conditions

The bacterial strains and plasmids used in this study, along with their relevant characteristics, are described in Table S1. The primers used in this study are listed in Table S2. The *E. miricola* FL160902 (wild-type, WT) was isolated from an infected frog during the 2016 outbreak in Hunan, China [10]. The strains of *E. miricola* or *Escherichia coli* (*E. coli*) were cultured in Brain-Heart Infusion (BHI) broth (Hopebio, Qingdao, China) or on BHI agar plates at 37°C. For the selective growth of bacterial strains, the medium was supplemented with appropriate antibiotics (Biosharp, Guangzhou, China) at the following concentrations: ampicillin (Amp), 100 μg/mL, and erythromycin (Erm), 50 μg/mL.

### Identification and analysis of genes encoding OmpA proteins

The nucleotide sequences of *ompA* genes in *E. miricola* FL160902 (NCBI accession: NZ_CP040516.1) were obtained from the National Center for Biotechnology Information (NCBI) database (https://www.ncbi.nlm.nih.gov). The genomic loci of these genes were mapped using TBtools-II software. To evaluate the conservation and distribution of these OmpA proteins across the *Elizabethkingia* genus, each deduced amino acid sequence was used as a query in BLASTP searches against a custom database. This database comprised all 437 *Elizabethkingia* genomes available in the NCBI RefSeq repository at the time of analysis, representing the complete set of publicly sequenced strains across different *Elizabethkingia* species. Homologous proteins were identified based on a threshold of ≥80% amino acid sequence identity. Genomic coverage was then calculated as the percentage of strains harboring such homologous proteins. Sequence comparisons were performed using the NCBI BLAST web interface (http://blast.ncbi.nlm.nih.gov).

### Construction of the ompA mutants and complemented strains

The *ompA* mutants were generated via homologous recombination as previously described [35]. For each gene, the upstream and downstream regions were amplified from *E. miricola* FL160902 genomic DNA using the primer sets listed in Table S2. The suicide plasmid pYT354 was digested with Xba I and Sac I to generate a linear vector. The purified flanking fragments for each *ompA* gene were then individually ligated into the linearized pYT354 vector to generate recombinant plasmids, which were subsequently transformed into competent *E. coli* S17-1 λpir cells. For conjugation, the donor strain (*E. coli* S17-1 λpir harboring a recombinant plasmid) and the recipient strain (*E. miricola* FL160902) were grown in BHI broth overnight. Cells from the donor and recipient cultures were mixed, spotted onto a Millipore filter membrane (0.22 μm), and incubated on a BHI agar plate at 37°C for 24 h. Transconjugants were first selected on BHI plates supplemented with Erm (50 μg/mL) and then subjected to counterselection on BHI plates containing 10% sucrose. Putative mutant colonies were verified by PCR amplification and DNA sequencing.

The complemented strains were constructed by integrating each *ompA* gene into its respective mutant strain’s chromosome at a highly conserved region (neutral site), employing homologous recombination as previously described [36]. Briefly, the upstream and downstream regions of the neutral site, as well as each *ompA* coding sequence, were amplified from the *E. miricola* FL160902 genome with the primers specified in Table S2. The *ompA* gene promoter fragment (114 bp) was artificially synthesized by Shenggong Biotechnology Co., Ltd. (Shanghai, China). The pYT354 vector was linearized by digestion with Kpn I and Sac I. Subsequently, the promoter fragment, the *ompA* coding sequence, and the neutral site flanking fragments were assembled and ligated into the linearized pYT354 backbone to generate knock-in plasmids. These plasmids were then introduced into their corresponding mutant strains via conjugation, following a procedure analogous to the knockout method.

### Bacterial growth curve assays and colony pigmentation analysis

The WT, the mutants, and the complemented strains were cultured overnight and diluted 1:100 in BHI broth. The bacterial suspensions were transferred into 24-well plates (Corning, NY, USA) containing 1.5 mL per well, with four wells for each culture. The cultures were incubated at 37°C with shaking at 350 rpm. The OD_600_ values were measured using a FLUOstar Omega microplate reader (BMG LABTECH, Germany) every hour for 24 hours. Three independent biological replicates were measured. For colony pigmentation analysis, 10 mL of each culture (WT, mutants, and complemented strains) was washed three times with PBS, resuspended, and spotted onto white filter paper. The samples were photographed using a fixed camera setup with consistent illumination and a uniform background. The mean grayscale values of the colonies were quantified using ImageJ software. The experiment was repeated three times independently.

### Stress resistance assay

The WT, the mutants, and the complemented strains were cultured in BHI broth to the stationary phase and resuspended in PBS at a density of 1 × 10^7^ CFU/mL. To evaluate stress tolerance, bacterial cells were subjected to oxidative and desiccation stress. For oxidative stress, cultures were exposed to 10 mM H_2_O_2_ for 60 min at 37°C. For desiccation stress, bacterial suspensions were spotted onto polycarbonate membranes and incubated at 37°C for 30 and 60 min. Following each stress treatment, cells were resuspended, serially diluted, and plated onto BHI agar to enumerate viable bacteria. The survival rate was calculated using the following formula: bacterial survival rate = (viable count after stress/viable count before stress) × 100%. The data were obtained from three independent experiments, and each experiment was performed in triplicate.

### Bacterial biofilm and hydrophobicity assays

Biofilm formation was assessed using crystal violet staining as previously described with minor modifications [37]. Briefly, the WT, the mutants, and the complemented strains were cultured overnight in BHI broth at 37°C and then adjusted to an OD_600_ of 1.0. The bacterial cultures were diluted 1:100 in fresh BHI broth, and 200 μL of each suspension was transferred to a 96-well plate for incubation at 37°C for 48 hours. The plate was washed three times with phosphate-buffered saline (PBS) to remove unattached bacteria and stained with 0.5% crystal violet (Solarbio, Beijing, China) for 30 min at room temperature. The staining solution was removed, and the plate was washed three times with PBS. After removing the washing solution, 200 μL of 95% ethanol was added to each well to dissolve the biofilm-bound crystal violet. The OD_590_ was obtained as an index of biofilm formation. All assays were carried out in triplicate and repeated on three separate occasions to confirm reproducibility.

The hydrophobicity assay was performed as follows. Briefly, the WT, the mutants, and the complemented strains were cultured to the logarithmic phase, washed three times, and resuspended in PBS to a density of 1 × 10^8^ CFU/mL. Then, 6 mL of the cell suspension in PBS was mixed thoroughly with 2 mL of octane for 2 min, and the initial OD_6__00_ (ODI) was recorded. The OD_6__00_ of the aqueous phase was measured after 30 min (ODF). The percentage of cell hydrophobicity was calculated as follows: [1-(ODF/ODI)] × 100%.

### Serum killing assay

The susceptibility of the WT, the mutants, and the complemented strains to serum‑mediated killing was determined as described previously with slight modifications [38]. Approximately 5 mL of blood was collected from healthy frogs and centrifuged at 3000 rpm for 10 min to obtain normal serum. Sera from multiple individuals were pooled to obtain sufficient volume. Normal serum was treated at 56°C for 30 min to inactivate the complement, generating heat-inactivated serum. Both sera were filter-sterilized (0.22 μm), and aliquots were stored at −80°C. Briefly, bacteria were cultured overnight and adjusted to a cell density of 1 × 10^8^ CFU/mL. The bacterial cells were washed with sterile PBS three times and resuspended in PBS. The normal and inactivated sera (100 μL) were separately mixed with 100 μL bacterial suspension to achieve a final concentration of 50% serum. Additionally, normal and inactivated sera (180 μL) were separately mixed with 20 μL bacterial suspension to achieve a final concentration of 90% serum. When the mixtures were incubated at 28°C for 60 min, the reaction mixtures were 10-fold serially diluted and plated onto BHI agar plates. The plates were incubated at 37°C for 48 h, and the colonies were counted. Survival rate was calculated as follows: (number of colonies with normal serum/number of colonies with heat-inactivated serum)×100%. Experiments for each strain were performed in triplicate.

### Bacterial adherence and anti-phagocytosis assays

The adherence and anti-phagocytosis assays were performed as previously described [39]. Adhesion experiments were conducted using the EPC cell line (epithelioma papulosum cyprini), and anti-phagocytosis assays were performed using the RAW264.7 cell line (murine monocyte macrophage). The cells were incubated in 12-well cell culture plates to 90% confluence. The WT, the mutants, and the complemented strains were cultured overnight at 37°C, standardized to an optical density (OD_600_) of 1.0 and subsequently washed three times with sterile PBS. The cells in each well were infected with the tested strains at an MOI (multiplicity of infection) of 20 and then incubated for 3 hours at 37°C. Non-adherent bacteria were removed by rinsing the wells three times with PBS, and the cells were released from the plate with 1% Triton X-100 (Solarbio, Beijing, China) for 30 min. The cell suspension was serially diluted 10-fold with PBS and spread onto BHI agar plates to count the number of viable bacteria.

Anti-phagocytosis assay was similarly performed to that of adhesion assay, except that the infected cells were incubated with DMEM containing gentamicin (100 μg/ml) for another 1 h to kill the surface and extracellular adhered bacteria. Following gentamicin treatment, the cells were washed three times with PBS to remove residual antibiotics and then lysed for enumeration of internalized bacteria. All samples were tested in triplicate, and experiments were repeated three times.

### Animal experiment

All black-spotted frogs (*Pelophylax nigromaculatus*) weighing 25 ± 0.5 g used in this study were obtained from a frog farm (Qianjiang, Hubei, China). Prior to experimentation, the frogs were acclimated in our facility for 2 weeks under controlled temperature conditions (28–30°C). Overnight bacterial cultures were washed three times with PBS and adjusted to a uniform cell density of 1 × 10^8^ CFU/mL. Healthy frogs were randomly allocated into six groups (*n* = 20 per group). Frogs were anesthetized by immersion in 1 g/L MS-222 (tricaine methanesulfonate) and then inoculated intramuscularly in the inner thigh with 100 µL of either WT or mutant bacterial strains, following a previously described procedure [36]. Frogs in the negative control groups received an equal volume of sterile PBS. Clinical signs of infection were monitored daily for 2 weeks, and mortality was recorded. At the end of the observation period, all remaining frogs were euthanized by immersion in 5 g/L MS-222 for 60 min [40]. The survival curve analysis was calculated using the Kaplan–Meier method.

### Statistical analyses

All statistics were performed using GraphPad Prism 7 software package (GraphPad Software, San Diego, CA). Numerical data were expressed as means ± SD and evaluated with a one-way analysis. *p* values of <0.05 were considered statistically significant.

## Results

### Identification of ompA genes in *E. miricola* FL160902

**Figure 1.**
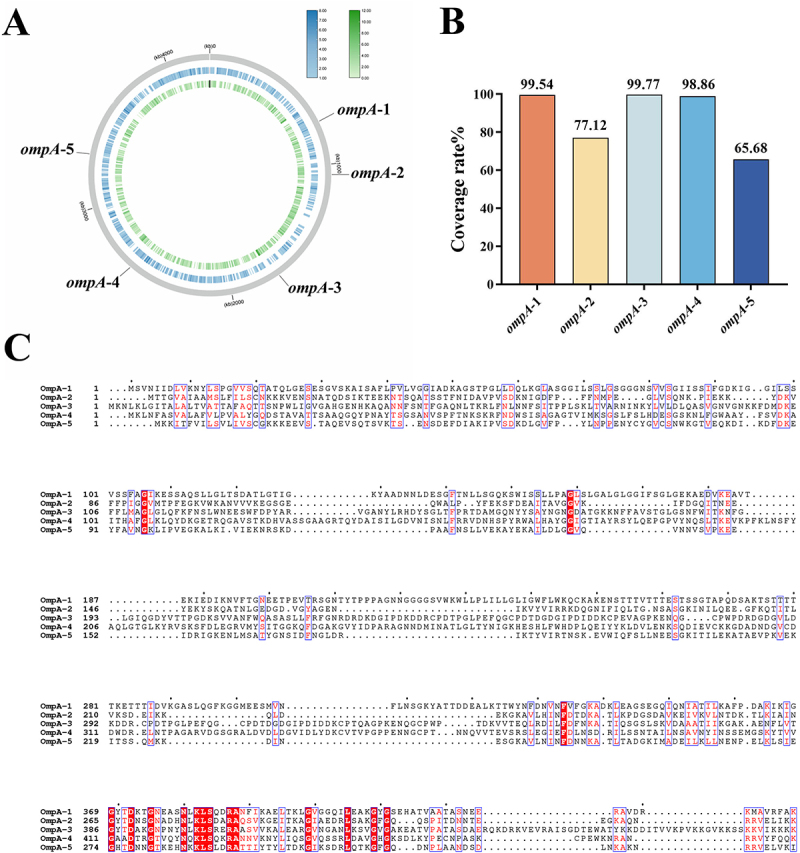
Identification of the five *ompA* genes in *E. miricola* strain FL160902. (A) circular genome map of *E. miricola* FL160902 showing the locations of five *ompA* genes. The inner concentric circles depict the density of forward-strand (green) and reverse-strand (blue) genes along the chromosome. The color gradient scales on the right correspond to gene density levels; (B) coverage rates of five *ompA*e gene homologs (>80% amino acid identity) across 437 publicly available *Elizabethkingia* genomes; (C) multiple alignments of translated sequences of *ompA* genes in *E. miricola* strain FL160902. The red highlight indicates identical amino acids among the tested OmpA proteins. OmpA-1 (accession number: QHQ85886.1), OmpA-2 (QHQ86140.1), OmpA-3 (QHQ86667.1), OmpA-4 (QHQ87428.1), OmpA-5 (QHQ88308.1).

Genomic analysis of *E. miricola* FL160902 identified five *ompA* genes, designated as *ompA*-1 (FE632_RS03365), *ompA*-2 (FE632_RS04790), *ompA*-3 (FE632_RS07740), *ompA*-4 (FE632_RS11825), and *ompA*-5 (FE632_RS15060), their chromosomal loci are annotated in Figure 1(A). Comparative analysis of 437 publicly available *Elizabethkingia* genomes revealed distinct patterns of conservation among the five OmpA proteins: OmpA-1, OmpA-3, and OmpA-4 were broadly conserved, with coverage rates of 99.54% (435/437), 99.77% (436/437), and 98.86% (432/437), respectively, whereas OmpA-2 and OmpA-5 exhibited lower coverage rates of 77.12% (337/437) and 65.68% (287/437), respectively (Figure 1(B)). Multiple sequence alignment further demonstrated high sequence conservation within the C-terminal domains across all OmpA proteins (Figure 1(C)).

### Identification of ompA mutants and complementation strains

**Figure 2.**
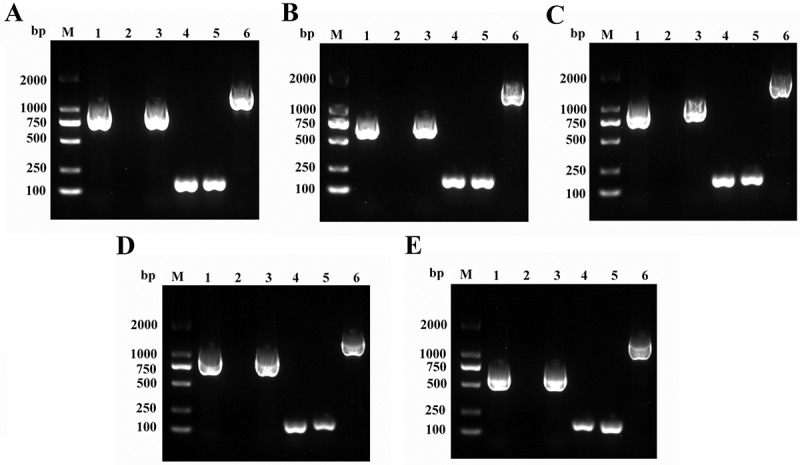
Identification of *ompA* mutants and complement strains by PCR amplification. (A-E) PCR identification of Δ*ompA*-1 (A), Δ*ompA*-2 (B), Δ*ompA*-3 (C), Δ*ompA*-4 (D), Δ*ompA*-5 (E), respectively. Lane M indicates the DL 2000 DNA Marker. Lanes 1and 4 are the amplified products of the WT strain, lanes 2 and 5, are the amplified products of mutants, lanes 3 and 6 are the amplified products of complement strains; the primers of lanes 1–3 are *ompA*_FL160902_-F/R, the primers of lanes, the primers of lanes 4–6 are Check-knockin-F/R.

To confirm the identity of the mutant and complementation strains, PCRs with different primer sets were performed, followed by DNA sequencing. As shown in Figure 2(A–E), the primers *ompA*_*FL160902*_-F/R did not generate a product for the mutant strains (Lane 2) but did produce the expected fragments for both the WT and complemented strains (Lanes 1 and 3), indicating successful disruption of the target *ompA* sequence in the mutants. Conversely, PCR using external primers targeting the insertion site (Check-F/R) yielded distinct products for the complemented strains (lane 6) compared to both the WT and mutant strains (lanes 4 and 5), confirming the successful chromosomal integration of each *ompA* gene into its respective mutant background.

### OmpA-1, OmpA-4, and OmpA-5 promote bacterial pigmentation

**Figure 3.**
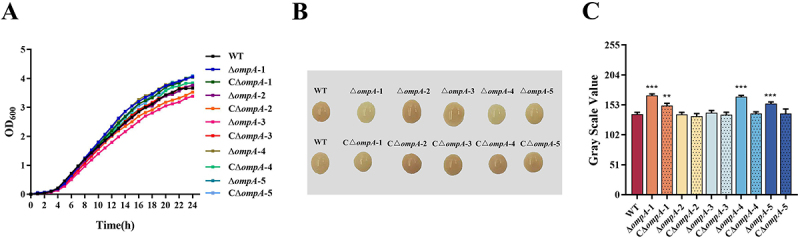
The growth curve and colony pigmentation of the WT, *ompA* mutants, and complement strains. (A) Growth of all the strains cultured at 37°C in BHI broth. Cell density was measured spectrophotometrically at a wavelength of 600 nm. (B) Bacterial pigmentation of the WT, *ompA* mutants, and complement strains. Error bars indicate the standard deviation from three independent tests. (C) Grayscale values of the WT, ompA mutants, and complementation strains. Error bars indicate the standard deviation from three independent tests. Asterisks indicate statistically significantly different from WT strain, **p < 0.01,***p < 0.001.

To evaluate the effects of each *ompA* mutation on bacterial growth, the WT, mutant, and complemented strains were cultured in BHI broth until they reached the stationary phase (Figure 3(A)). The growth curves revealed no significant differences among the strains in BHI medium at 37°C, indicating that deletion of these OmpA proteins does not impair general growth. Moreover, colony morphology, including size, shape, elevation, and margin, did not differ significantly between the WT and any *ompA* mutant on BHI agar plates. However, distinct differences in colony pigmentation were observed. Specifically, the Δ*ompA*-1, Δ*ompA*-4, and Δ*ompA*-5 mutants all exhibited reduced pigmentation compared to the WT strain, with the Δ*ompA*-1 mutant displaying the most pronounced change (*p* < 0.001; Figure 3(B,C)). These findings suggest that OmpA-1, OmpA-4, and OmpA-5 contribute to pigment deposition, likely by participating in its biosynthesis, regulation, or transport.

### OmpA-1 and OmpA-3 enhance oxidative stress resistance in *E.Mircola*

**Figure 4.**
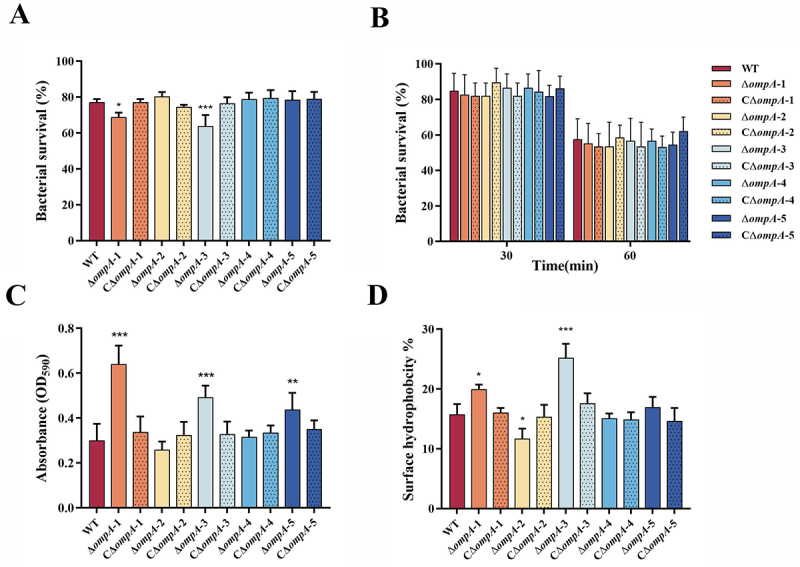
Phenotypic characteristics of the WT, *ompA* mutants and complement strains. (A) comparison of survival rates between the WT, *ompA* mutants and complement strains under H_2_O2 conditions. (B) the survival rates between the WT, *ompA* mutants and complement strains under desiccation conditions. (C) the biofilm formation capacities of the WT, *ompA* mutants and complement strains. (D) the percentages of cells into the hydrophobic solvent octane; error bars represent mean ± standard error. Asterisks indicate statistically significantly different from WT strain, *p < 0.05, **p < 0.01, ***p < 0.001.

To determine whether OmpA proteins contribute to stress tolerance, we evaluated the growth of *ompA* mutants under oxidative and desiccation stress. When exposed to H_2_O_2_, the Δ*ompA*-1 and Δ*ompA*-3 mutants showed significantly increased sensitivity compared to the WT strain (Figure 4(A)). The relative survival rates of the Δ*ompA*-1 and Δ*ompA*-3 strains were 68.98% (*p* < 0.05) and 63.84% (*p* < 0.001), respectively, whereas the WT strain survival rate was 77.23%. In the complemented strains, oxidative stress resistance was restored to WT levels. By contrast, no significant difference in survival was observed between any of the mutants and the WT under desiccation stress (Figure 4(B)).

### OmpA-1 and OmpA-3 are key negative regulators of biofilm formation and surface hydrophobicity

Biofilm formation by the WT, mutant, and complemented strains was quantified using crystal violet staining. As shown in Figure 4(C), the Δ*ompA*-1, Δ*ompA*-3, and Δ*ompA*-5 mutants all exhibited a significant increase in biofilm biomass (OD_5__90_) compared to the WT strain (*p* < 0.001). Notably, the enhancement was most pronounced in the Δ*ompA*-1 and Δ*ompA*-3 mutants, indicating that OmpA-1 and OmpA-3 play a predominant role in negatively regulating biofilm development. Subsequently, the hydrophobic properties of *ompA* mutants were assessed. A statistically significant increase in surface hydrophobicity was observed only for the Δ*ompA*-1 and Δ*ompA*-3 mutants (Figure 4(D)), which is consistent with the increased biofilm formation. In contrast, the Δ*ompA*-2 mutant showed reduced hydrophobicity relative to the WT, while no significant changes were detected in the remaining mutants. Complementation of each respective mutant restored surface hydrophobicity to levels comparable to the WT.

### OmpA-1 and OmpA-3 are essential determinants of serum resistance

**Figure 5.**
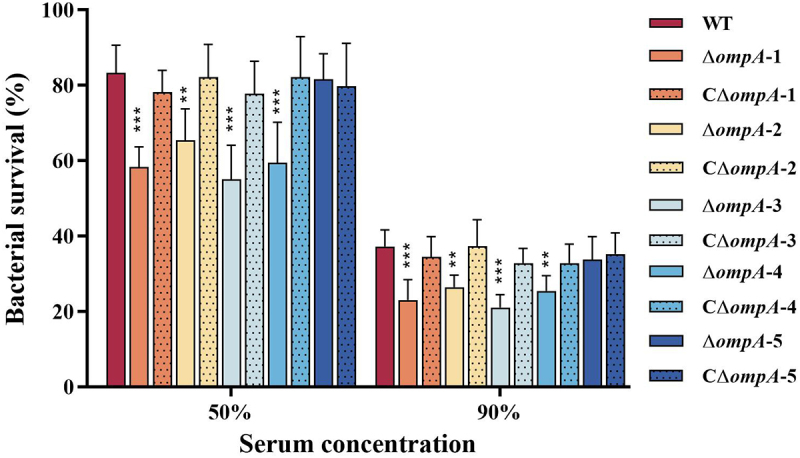
Serum killing assay of WT, *ompA* mutants and complement strains. The percent survival was calculated as the ratio of bacterial colonies survived in normal serum (NS) divided by the colonies survived in heat-treated serum (HS). Error bars indicate the standard deviation from three independent tests. Asterisks indicate statistically significantly different from WT strain, **p < 0.01,***p < 0.001.

To evaluate the contribution of OmpA proteins to serum resistance, serum killing assays were performed. Following exposure to 50% or 90% frog serum, all *ompA* mutants except Δ*ompA*-5 exhibited significantly reduced survival compared to the WT strain (Figure 5). A hierarchy of susceptibility was observed among the mutants: Δ*ompA*-3 > Δ*ompA*-1 > Δ*ompA*-4 > Δ*ompA*-2. Specifically, in 50% serum, the survival rates were 55.13%, 58.33%, 59.52%, and 65.48% for the mutants, respectively, versus 83.33% for the WT. A corresponding reduction was evident in 90% serum, with mutant survival rates of 21.08%, 23.08%, 25.49%, and 26.47%, compared to 37.26% for the WT. Genetic complementation of each respective mutant fully restored serum tolerance to the WT level. Taken together, these results establish OmpA-1 and OmpA-3 as essential mediators of serum resistance.

### OmpA-3 is critical for adhesion and anti-phagocytosis ability in E. miricola

**Figure 6.**
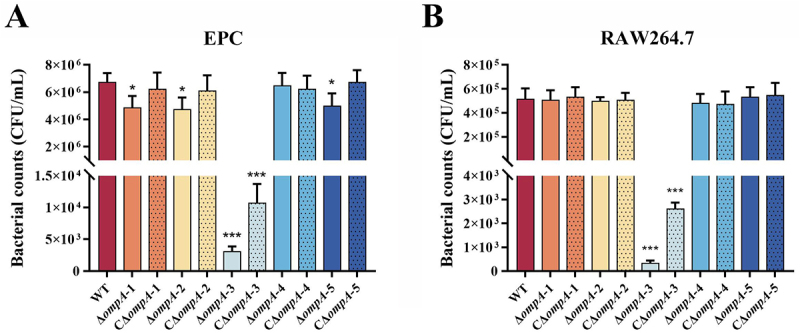
Adherence and anti-phagocytosis assays for the WT, *ompA* mutants and complement strains. (A) adherence assay was performed on EPC cells. (B) anti-phagocytotic assay was performed on in macrophage RAW264.7 cells. The data represent the number of bacteria bound to or invaded into cells in each well of 6-well plate. The error bars represent means ± standard deviations from three independent experiments. Asterisks indicate statistically significantly different from WT strain, *p < 0.05, ***p < 0.001.

The roles of OmpA proteins in bacterial adhesion and resistance to phagocytosis were systematically investigated using epithelial cells (EPC) and macrophages (RAW 264.7). As shown in Figure 6(A), adhesion assays revealed significantly reduced cell adhesion in all *ompA* deletion mutants compared to the WT strain (*p* < 0.05), except for the Δ*ompA*-4 mutant. Strikingly, the adhesion capacity of the Δ*ompA*-3 mutant was reduced by 3 orders of magnitude compared to the WT strain (*p* < 0.001). Partial restoration of adhesion was observed in the *ompA*-3 complemented strain, though levels remained significantly lower than those of the WT (*p* < 0.001), implicating OmpA-3 as a critical mediator of bacterial adherence. Conversely, phagocytosis assays yielded a divergent phenotype for the Δ*ompA*-3 mutant. As shown in Figure 6(B), it demonstrated a significantly enhanced resistance to phagocytosis, with an approximately 1000-fold decrease in the number of bacterial cells internalized by macrophages compared to the WT strain (*p* < 0.001). Genetic complementation of Δ*ompA*-3 partially reduced this elevated resistance, yet the strain did not revert to full WT susceptibility (*p* < 0.001).

### OmpA proteins differentially contribute to virulence in a frog infection model

**Figure 7.**
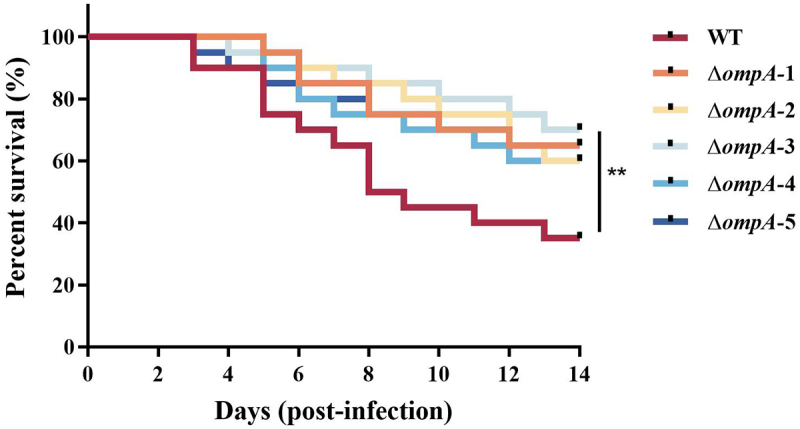
Survival curves of frogs infected with the WT or *ompA* mutants. *n* = 20 for the WT and mutant strains infected groups. Asterisks indicate statistically significantly different from WT strain, **p < 0.01.

To assess the role of OmpA proteins in *E. miricola* pathogenesis, we compared the virulence of WT and mutant strains in a frog infection model. Infected frogs exhibited a range of clinical signs, including ascites, cataracts, and neurological manifestations. All mutant strains exhibited significantly attenuated *in vivo* pathogenicity compared to the WT, as evidenced by markedly higher survival rates of infected frogs (Figure 7). Specifically, frogs infected with the Δ*ompA*-1 (65%), Δ*ompA*-2 (60%), Δ*ompA*-3 (70%), Δ*ompA*-4 (60%), and Δ*ompA*-5 (65%) mutants showed 1.86-, 1.71-, 2.00-, 1.71-, and 1.86-fold increases in survival, respectively, relative to the WT strain (35% survival; *p* < 0.01). These findings conclusively demonstrate that OmpA proteins are critical for the *in vivo* pathogenicity of *E. miricola* FL160902.

## Discussion

**Figure 8.**
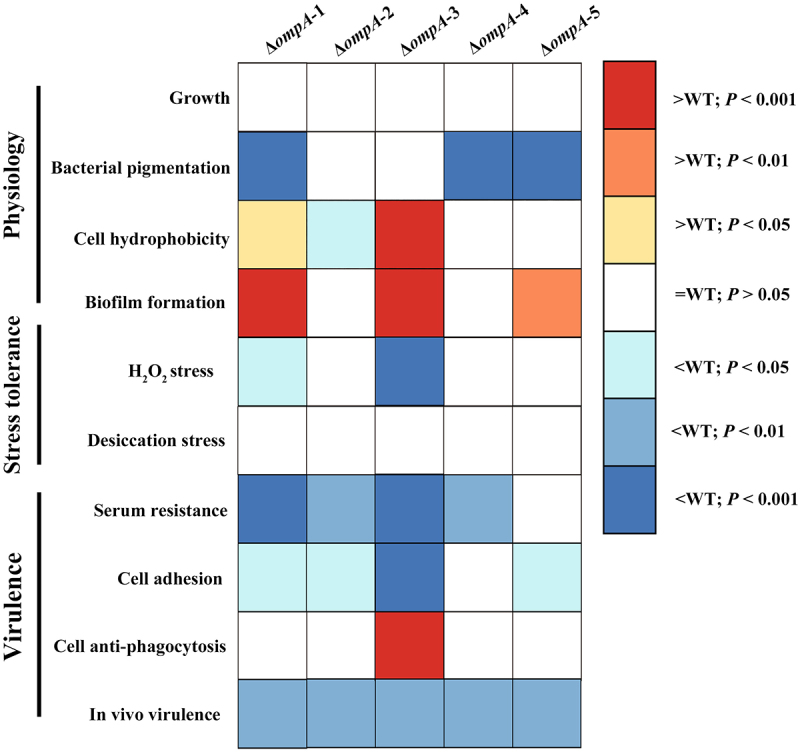
Heat map representing the functional characterization of *E. miricola* strain FL160902 OmpA proteins. The role of each gene in bacterial physiology, stress tolerance, and virulence was assessed by different methods, as previously described. The color code represents the relationship with WT data, based on *p* values, with blue hues indicating that mutants were more defective and orange hues indicating that mutants were more proficient compared to the WT for each specific feature. The intensity of the color is directly proportional to the statistical significance value. White boxes indicate no statistical difference compared to the WT strain.

The increasing prevalence of multidrug-resistant *Elizabethkingia* species underscores a critical and urgent need for innovative therapeutic strategies. OmpA family proteins represent promising targets for such development due to their well-established roles in bacterial pathogenicity. This study provides the first comprehensive functional characterization of the OmpA protein family in *E. miricola* (summarized in Figure 8), revealing a striking functional divergence among its five paralogs and establishing a clear functional hierarchy, with OmpA-1 and OmpA-3 identified as master regulators of virulence and key physiological processes.

A central finding of this study is the identification of OmpA-1 and OmpA-3 as pivotal, non-redundant orchestrators of virulence, with their profound deletion phenotype being mechanistically explained by their multifaceted roles in host invasion and immune subversion. A primary fitness function is serum resistance, a critical prerequisite for systemic dissemination [41]. Our data demonstrate that OmpA-1 and OmpA-3 significantly enhance this trait, aligning with the known role of OmpA homologs in other Gram-negative pathogens, which evade complement-mediated killing by recruiting host regulators [30,42]. Based on this functional parallel, we propose these effectors likely subvert the complement cascade through specific, essential interactions with host regulatory components, thereby augmenting serum resistance in *E. miricola*. Furthermore, OmpA-3 emerges as a primary adhesion, where its deletion causes a dramatic, multi-log reduction in epithelial cell adhesion, positioning it as a key mediator of the initial host-pathogen encounter. Interestingly, deletion of *ompA*-3 resulted in increased resistance to phagocytosis. Phagocytic clearance relies on direct or indirect recognition by phagocyte surface receptors. We hypothesize that OmpA-3 serves as a direct ligand for macrophage phagocytic receptors and that its absence may therefore diminish macrophage-mediated recognition and internalization, thereby promoting bacterial immune evasion [43]. The incomplete phenotypic restoration observed upon genetic complementation of the Δ*ompA*-3 mutant is likely multifactorial, potentially stemming from insufficient transcriptional activity of the exogenous promoter relative to the native regulatory context, resulting in subphysiological protein abundance, or from improper folding or mislocalization of the ectopically expressed protein, thereby precluding full functional recovery. Notably, while OmpA-1 and OmpA-3 dominate the virulence profile, the significant attenuation observed *in vivo* infection models for all single mutants, including Δ*ompA*-2, Δ*ompA*-4, and Δ*ompA*-5, underscores that the OmpA suite operates as an integrated system in which each homolog contributes to the overall pathogenic fitness, suggesting a network of complementary, non-antagonistic functions.

This functional divergence extends beyond critical virulence traits into core physiological regulation, further highlighting the integrative role of OmpA-1 and OmpA-3. Paradoxically, they function as key negative regulators of biofilm formation and surface hydrophobicity. Their deletion triggers a hyper-biofilm, hyper-hydrophobic state, which in many contexts would be associated with enhanced persistence [44]. However, in our infection model, this state is correlated with reduced virulence, suggesting it may be a maladaptive stress response or a compensatory shift to a sessile lifestyle upon the loss of invasive capability [45,46]. Their role in oxidative stress resistance further solidifies their status as critical signal integrators. The increased susceptibility of the Δ*ompA*-1 and Δ*ompA*-3 mutants to H_2_O_2_ indicates that these proteins are essential for countering oxidative stress. We propose that OmpA-1 and OmpA-3 enhance bacterial tolerance to oxidative stress by stabilizing the outer membrane, a process likely mediated through non-covalent interactions between their flexible periplasmic domains and the underlying peptidoglycan layer [47]. This structural reinforcement helps preserve membrane integrity upon exposure to reactive oxygen species (ROS) and may also support the regulation of adaptive stress responses, thereby ensuring bacterial survival within the hostile host environment [23].

In contrast, the remaining paralogs exhibit more modular and specialized functionalities. Specifically, OmpA-4 and OmpA-5, together with OmpA-1, are involved in the modulation of pigmentation. Given that OmpA proteins can function as channels for small molecules [22], it is plausible that these three paralogs contribute to pigment transport or membrane anchoring, thereby influencing pigmentation. OmpA-2 uniquely modulates surface hydrophobicity in a manner opposite to OmpA-1 and OmpA-3, indicating a distinct role in fine-tuning envelope physicochemical properties. Furthermore, OmpA-5, along with OmpA-1 and OmpA-3, functions as a negative regulator of biofilm formation. Additionally, OmpA-2, OmpA-4, and OmpA-5 contribute variably, though less prominently, to specific virulence traits. This pattern of “master regulators” versus “specialized modules” is characteristic of functionally divergent gene families and is observed beyond *Elizabethkingia*. In *E. coli*, for example, while canonical OmpA maintains structural integrity and modulates immune responses, other OmpA family paralogs have acquired specialized roles in antibiotic resistance and stress adaptation [48,49]. Similarly, in *A. baumannii*, distinct Omp paralogs have evolved specificities toward resisting different classes of antimicrobial peptides [50,51]. This pattern supports a common evolutionary trajectory wherein gene duplication supplies the genetic substrate for functional diversification [52]. Subsequent selective pressures drive neofunctionalization or subfunctionalization, enabling bacteria to expand their adaptive repertoire and thrive in diverse or fluctuating niches without compromising essential core functions.

The molecular basis for this divergence in *E. miricola* can be explained by its protein architecture. The high conservation of the C-terminal domains across all five paralogs, which are responsible for peptidoglycan binding and fundamental membrane anchoring, preserves the shared structural scaffold [53,54]. Therefore, the profound phenotypic differences almost certainly emanate from the hyper-variable, solvent-exposed N-terminal extracellular loops. These loops act as specificity determinants, dictating unique interactions with diverse host components such as complement proteins, epithelial cell receptors, or signaling molecules [55]. Supporting this, our genomic analysis reveals differential evolutionary conservation: the genes encoding OmpA-1 and OmpA-3, under strong purifying selection due to their core virulence functions, exhibit high sequence conservation. In contrast, the genes for *ompA*-2 and *ompA*-5 are less conserved, potentially subject to diversifying selection for niche adaptation or harboring conditionally beneficial alleles, a feature that grants them greater sequence plasticity. This divergence likely represents a form of functional complementarity within the outer membrane proteome, wherein a stable, essential structural core is co-opted and diversified by variable loops to create an adaptable repertoire for multifaceted environmental interaction. Given the cooperative contribution of all paralogs to virulence, antagonistic interactions appear improbable; instead, they collectively constitute a synergistic functional network.

By delineating this functional hierarchy, we identify OmpA-1 and OmpA-3 as the highest-priority targets for anti-virulence therapeutics. Potential strategies include the design of monoclonal antibodies or engineered peptides that block their critical extracellular loops, thereby disrupting serum resistance, adhesion, or evasion of phagocytosis [19,56]. Such agents, used as adjuvants to conventional antibiotics, could disarm the pathogen, enhance host clearance, and potentially reduce the selective pressure driving traditional antibiotic resistance [57]. Furthermore, this study provides a clear roadmap for future research. Immediate priorities include elucidating the precise molecular mechanisms governing their function, defining the regulon controlled by OmpA-1 and OmpA-3, and determining high-resolution structures of these proteins to guide rational inhibitor design. Beyond this, exploring the potential cross-talk and regulatory interplay among the paralogs, especially under different environmental stresses, will reveal how this networked system dynamically responds to challenges.

In conclusion, this study significantly advances our understanding of the OmpA family in *Elizabethkingia*, shifting its functional profile from relative obscurity to a clarified model of hierarchical paralog specialization. It identifies OmpA-1 and OmpA-3 as promising molecular targets for therapeutic intervention. Consequently, these findings not only provide crucial insights into the pathogenesis of *Elizabethkingia* but also establish a foundational framework for developing targeted treatment strategies against this multidrug-resistant pathogen.

## Supplementary Material

Table S2.docx

Table S1.docx

## Data Availability

The data that support the findings of this study are available in this article, supplementary files, and F igshare (https://figshare.com/) with DOI: https://doi.org/10.6084/m9.f igshare.30892274 [58].
