# Supplementary material for: Divergent roles of OmpA family proteins in physiology, stress tolerance, and virulence of Elizabethkingia miricola
Source: Virulence. 2026 Apr 11;17(1):2645897. doi: 10.1080/21505594.2026.2645897 (PMC13078209; doi:10.1080/21505594.2026.2645897)
Supplement: Table S2.docx [file KVIR_A_2645897_SM6751.docx]

Table S2. Primers used in this study.

| Primer name | Sequence(5’-3’) | Target gene | Source or Reference |
| --- | --- | --- | --- |
| *ompA*-1-Up-F/R | CATGAATTCCCGGGAGAGCTCCACTCCGGAAGAAGAAGA  CAGAAGACGGCATACGAATACGCCAGGCGAAAGATA | The upstream of *ompA*-1 | This study |
| *ompA*-1-Down-F/R | AGAGTAGGGAACTGCCAGGCAACTGCATCTAACGAA  CGATCCCAAGCTTCTTCTAGATGAAATGTGCTGGCAGCC | The downstream of *ompA*-1 | This study |
| *ompA*-2-Up-F/R | CATGAATTCCCGGGAGAGCTCACTGATCGGCTACAGTAG  CAGAAGACGGCATACGAACCAAAGCCACACTAGCAA | The upstream of *ompA*-2 | This study |
| *ompA*-2*-*Down-F/R | AGAGTAGGGAACTGCCAGGAATGGAGCAATCTTCC  CGATCCCAAGCTTCTTCTAGAGTACCTATACCTCCGTAAG | The downstream of *ompA*-2 | This study |
| *ompA*-3-Up-F/R | CATGAATTCCCGGGAGAGCTCAACGGGTGTCCAGTGAAA  CAGAAGACGGCATACGAAACCATAAAGGGCAACAGG | The upstream of *ompA*-3 | This study |
| *ompA*-3-Down-F/R | AGAGTAGGGAACTGCCAGTCCTGCAAGCAAATGTCC  CGATCCCAAGCTTCTTCTAGACTGCTCTATGCCGCTAAT | The downstream of *ompA*-3 | This study |
| *ompA*-4-Up-F/R | CATGAATTCCCGGGAGAGCTCCATTCCGGTTGCAGCTGTT  CAGAAGACGGCATACGAACTCCTGAGCTGTTGATAC | The upstream of *ompA*-4 | This study |
| *ompA*-4-Down-F/R | AGAGTAGGGAACTGCCAGGCGAAGAACAGAAGGGTAG  CGATCCCAAGCTTCTTCTAGAATCAAATCCTGGGAACTGC | The downstream of *ompA*-4 | This study |
| *ompA*-5-Up-F/R | CATGAATTCCCGGGAGAGCTCGATATTTTCGCTCCGGGT  CAGAAGACGGCATACGAACTGACAGGAATAAAGCTG | The upstream of *ompA*-5 | This study |
| *ompA*-5-Down-F/R | AGAGTAGGGAACTGCCAGCAGGATGCTAAACAAGGT  CGATCCCAAGCTTCTTCTAGAGCCATAACAGCATTGTAG | The downstream of *ompA*-5 | This study |
| neutral site-Up-F/R | TACTCAGAGCTCGGTGGAGCACCTATGT  TACTCAGGATCCTCGTATGAGGGCTCTT | Upstream fragment of neutral site | This study |
| neutral site-Downp-F/R | TACTCAGTCGACGGGCTCTGTAATAATTCGGG  TACTCAGGTACCGGAACCCCTGCTCAAA | Downstream fragment of neutral site | This study |
| PompA-F/R | AGCCCTCATACGAGGATCCCTTGCCACATTTGGTG  CAGTTCTTCGCCTTTGCTC | *ompA* gene promoter | This study |
| *ompA*-1-F/R | CTACTCAGCTTGGAGAAAG  ATTTGTCCGCCTACACCTA | External sequence of *ompA*-1 | This study |
| *ompA*-2-F/R | ATGGACAGGATTCCACGG  CCGCCATTCTTTCAGCATC | External sequence of *ompA*-2 | This study |
| *ompA*-3-F/R | TGCTACAACATCTGCAGC  CACTCTTACCATGTCCTAC | External sequence of *ompA*-3 | This study |
| *ompA*-4-F/R | GTTTCCTTACCTGAATCCG  GCAGCCAAAGGATTATCC | External sequence of *ompA*-4 | This study |
| *ompA*-5-F/R | GATATTTTCGCTCCGGGT  CTCTACTCTTCTGTTCTG | External sequence of *ompA*-5 | This study |
| *ompA*-1-in-F/R | CTACTCAGCTTGGAGAAAG  ATTTGTCCGCCTACACCTA | An internal region of *ompA*-1 | This study |
| *ompA*-2-in-F/R | ATGGACAGGATTCCACGG  CCGCCATTCTTTCAGCATC | An internal region of *ompA*-2 | This study |
| *ompA*-3-in-F/R | TGCTACAACATCTGCAGC  CACTCTTACCATGTCCTAC | An internal region of *ompA*-3 | This study |
| *ompA*-4-in-F/R | GTTTCCTTACCTGAATCCG  GCAGCCAAAGGATTATCC | An internal region of *ompA*-4 | This study |
| *ompA*-5-in-F/R | GATATTTTCGCTCCGGGT  CTCTACTCTTCTGTTCTG | An internal region of *ompA*-5 | This study |
| Check-knockin-F/R | GATGGCGTTTTAATGCAGGC  AAAACCGCTCATGGTGAGC | External sequence of neutral site | This study |
