# Supplementary material for: Divergent roles of OmpA family proteins in physiology, stress tolerance, and virulence of Elizabethkingia miricola
Source: Virulence. 2026 Apr 11;17(1):2645897. doi: 10.1080/21505594.2026.2645897 (PMC13078209; doi:10.1080/21505594.2026.2645897)
Supplement: Table S1.docx [file KVIR_A_2645897_SM6750.docx]

Table S1. Bacterial strains and plasmids used in this study

| Strains/plasmids | | Description | Source or Reference | |
| --- | --- | --- | --- | --- |
| Strains |  | | |  |
| FL160902 | Isolated from an infected frog during outbreak in Hunan, China. | | | Laboratory collection |
| △*ompA*-1 | *ompA*-1 deletion mutant of FL160902 strain | | | This study |
| △*ompA*-2 | *ompA*-2 deletion mutant of FL160902 strain | | | This study |
| △*ompA*-3 | *ompA*-3 deletion mutant of FL160902 strain | | | This study |
| △*ompA*-4 | *ompA*-4 deletion mutant of FL160902 strain | | | This study |
| △*ompA*-5 | *ompA*-5 deletion mutant of FL160902 strain | | | This study |
| C△*ompA*-1 | *ompA*-1 complemented strain of △*ompA*-1 strain | | | This study |
| C△*ompA*-2 | *ompA*-2 complemented strain of △*ompA*-2 strain | | | This study |
| C△*ompA*-3 | *ompA*-3 complemented strain of △*ompA*-3 strain | | | This study |
| C△*ompA*-4 | *ompA*-4 complemented strain of △*ompA*-4 strain | | | This study |
| C△*ompA*-5 | *ompA*-5 complemented strain of △*ompA*-5 strain | | | This study |
| *E. coli* S17-1 | hsdR17 recA1 RP4-2-tet::Mu-1kan::Tn7, SmR, Conjugal donor for recombinant vector | | | Laboratory collection |
| Plasmids |  | | |  |
| pYT354 | Erm^R^, Amp^R^, SacB suicide vector | | | [35] |

R , Resistance.
